# AMPK Activation Mediated by Hinokitiol Inhibits Adipogenic Differentiation of Mesenchymal Stem Cells through Autophagy Flux

**DOI:** 10.1155/2018/2014192

**Published:** 2018-07-10

**Authors:** Ju-Hee Lee, Jae-Kyo Jeong, Sang-Youel Park

**Affiliations:** ^1^Biosafety Research Institute, College of Veterinary Medicine, Chonbuk National University, Iksan, Jeonbuk 54596, Republic of Korea; ^2^New Drug Development Center, Daegu-Gyeongbuk Medical Innovation Foundation, 88 Dongnae-ro, Dong-gu, Daegu City 41061, Republic of Korea

## Abstract

**Background and Purpose:**

Hinokitiol, a natural monopenoid present in the essential oil of *Calocedrus formosana* heartwood, exerts potent anticancer, anti-inflammatory, antibacterial, and neuroprotective effects on various cells. However, the antiobesity effect of hinokitiol on adipocytes is unclear.

**Experimental Approach:**

In this study, we observed that hinokitiol affected the differentiation to adipocytes in mesenchymal stem cells (MSCs). Hinokitiol was treated with 3-isobutyl-1-methylxanthine, insulin, and dexamethasone to induce differentiation and maturing adipocytes in cultured MSCs.

**Key Results:**

Hinokitiol treatment of MSCs decreased their differentiation to mature adipocytes and increased AMPK phosphorylation in a concentration-dependent manner. Moreover, we confirmed that the antiadipogenic effect of hinokitiol was associated with autophagy. The levels of LC3-II decreased and those of p62 increased in hinokitiol-treated MSCs. The treatment of hinokitiol-treated MSCs with the autophagy activator, rapamycin, restored the hinokitiol-induced decrease in the adipocyte differentiation of MSCs. The inhibition of AMPK phosphorylation also suppressed hinokitiol-mediated inhibition of autophagy and antiadipogenic effects.

**Conclusions and Implications:**

Taken together, these results indicated that AMPK activation and autophagy flux inhibition mediated by hinokitiol inhibited lipid accumulation and differentiation of MSCs to adipocytes and also suggest that differentiation of mesenchymal stem cells may be regulated by using the modulator of autophagy flux and AMPK signals including hinokitiol.

## 1. Introduction

In recent times, the occurrence of obesity has increased worldwide [1]. Obesity is the key contributor to and a risk factor of various chronic diseases such as hypertension, type 2 diabetes, fatty liver disease, atherosclerosis, degenerative disorders, airway disease, and some cancers [2]. The incidence of obesity has increased because of changes in lifestyle and diet. A recent statistical report of the World Health Organization stated that one out of 10 adults was overweight, with more than one billion overweight adults worldwide.

Adipogenesis involves hyperplastic transformation of undifferentiated preadipocytes into mature adipocytes. Adipogenesis also involves the conversion of free fatty acids in the bloodstream to lipid droplets and their accumulation in adipocytes. It is also sequentially and cooperatively regulated by various transcription factors and adipocyte-specific genes such as CCAAT enhancer-binding proteins (C/EBP-*α* and C/EBP-*β*), peroxisome proliferator-activated receptor-*γ* (PPAR-*γ*), and aP2 [3–6]. C/EBP-*β* is expressed in the early stage of differentiation, and C/EBP-*α* and PPAR-*γ* are expressed in the late stage of differentiation. PPAR-*γ* regulates lipid and glucose homeostasis, insulin sensitivity, and endocrine function in the adipose tissue [7, 8]. C/EBP-*β*, which is activated by dexamethasone, promotes the expression of PPAR-*γ* and C/EBP-*α* [9, 10]. PPAR-*γ* and C/EBP-*α* regulate each other to promote their expression and to enhance adipogenesis [11]. Adipogenesis can be induced by treatment with insulin, dexamethasone, and 3-isobutyl-1-methylxanthine (IBMX) [12].

Autophagy is a major evolutionarily conserved degradation pathway for bulk cytoplasmic contents and subcellular organelles and is implicated in the formation of the adipose tissue [13, 14]. Autophagy is closely associated with adipogenesis and obesity [15–17]. Increased autophagy in adipose tissues in the case of obesity have also been shown in obese humans and animals [18–20]. Inhibition of autophagy-associated genes *Atg5* and *Atg7* significantly suppresses the adipogenesis of preadipocytes and attenuates diet-induced obesity in mice [15, 17].

AMP-activated protein kinase (AMPK) is the key inducer of autophagy [21] and plays an important role as the regulator of cellular and whole-body energy balance [22]. AMPK is a heterotrimeric enzyme composed of a catalytic *α* subunit and two regulatory *β* and *γ* subunits that are encoded by separate genes and that complex with each other to form 12 different isoforms [23]. AMPK promotes its phosphorylation [24] at the Thr172 residue in its *α* subunit [25] by using an upstream kinase that was identified as a complex between tumor suppressor protein LKB1 and two accessory subunits STRAD and MO25 [26]. AMPK regulates numerous proteins associated with nutrient metabolism by inhibiting anabolic ATP-consuming pathways and by activating catabolic ATP-generating pathways. During energy depletion, AMPK suppresses de novo fatty acid synthesis by inactivating acetyl-CoA carboxylase and stimulates fatty acid oxidation by enhancing the expression of carnitine palmitoyltransferase-1 and PPAR-*α* [27, 28]. AMPK suppresses the expression of C/EBP-*α*, C/EBP-*β*, C/EBP-*δ*, and PPAR-*γ* [29], and AMPK activator AICAR suppresses lipid metabolism by regulating *β*-oxidation-associated proteins during adipocyte differentiation [30]. These findings have considerably increased the interest in AMPK as a therapeutic target for treating metabolic dysfunction observed in persons with obesity and insulin resistance.

In the present study, we hypothesized that hinokitiol-induced reduction in lipid accumulation and triglyceride (TG) content in mesenchymal stem cells (MSCs) was mediated by the downregulation of C/EBP-*α* and PPAR-*γ* expression during adipogenesis through the AMPK pathway. The link between autophagy and AMPK during adipogenesis will also be further defined. Therefore, we suggested the possibility of hinokitiol as a target against obesity-related disease.

## 2. Materials and Methods

### 2.1. Cell Culture and Differentiation

MSCs were maintained in Dulbecco's modified Eagle's medium (DMEM) supplemented with 10% fetal bovine serum and antibiotics (100 *μ*g·mL^−1^ gentamicin and 100 *μ*g·mL^−1^ penicillin-streptomycin). Differentiation was induced by incubating 2-day postconfluent MSCs in an MDI induction medium (DMEM supplemented with 10% fetal bovine serum, 0.5 mM IBMX, 1 *μ*m dexamethasone, and 1 *μ*g/mL insulin) for 2 days. After 2 days, the induction medium was replaced by an insulin medium. Detection of AdipoRed assay was performed on day 7.

### 2.2. Quantification of Lipid Content

Lipid content was quantified using AdipoRed Assay Reagent (Lonza, Verviers, Belgium), according to the manufacturer's instructions. Briefly, preadipocytes grown in 24-well plates were incubated in the MDI medium alone or in the MDI medium supplemented with test compounds during the adipogenic phase and on day 7. Next, 300 *μ*L phosphate-buffered saline (PBS) was added to the wells, followed by the addition of 30 *μ*L AdipoRed reagent, and the cells were incubated for 10 min at 37°C. Fluorescence was measured at excitation and emission wavelengths of 485 and 572 nm, respectively.

### 2.3. Measurement of TG Content in Adipocytes

MSCs were harvested at 7 days after the induction of differentiation. After treatment of MDI, the medium was removed and cell extract was used for determining TG content. Matured cells were washed extensively with PBS, scraped on ice in 500 *μ*L sonication buffer (25 mM Tris buffer and 1 mM ethylenediaminetetraacetic acid (EDTA) (pH 7.5)), and sonicated to homogenize the cell suspension. Protein concentration was determined by lysing the cells in 0.3 N NaOH and 0.1% SDS and was measured using a BCA reagent. Total TG content in the cells was determined using a TG determination kit (T2449; Sigma-Aldrich). TG content results were obtained as glycerol/mg protein and were expressed as the ratio (%) to the control value.

### 2.4. Quantitative Reverse Transcription-Polymerase Chain Reaction

Total RNA was extracted from MSCs treated with hinokitiol by using Easy-spin™ total RNA extraction kit (iNtRON Biotechnology, Seoul, Korea). Next, cDNA was synthesized using PrimeScript™ 1st Strand cDNA Synthesis Kit (TaKaRa Bio, Tokyo, Japan), according to the manufacturer's instructions. Quantitative PCR (qPCR) was performed using a 1 *μ*L gene primer with SYBR Green (Bio-Rad Laboratories, Hercules, CA, USA) in a 20 *μ*L reaction volume. Sequences of primers used for performing qPCR are as follows: PPAR-*γ* forward, 5′CGGAAGCCCTTTGGTGACTTTATG3′; PPAR-*γ* reverse, 5′GCAGCAGGTTGTCTTGGATGTC3′ and C/EBP-*α* forward, 5′CGGGAACGCAACAACATCGC3′, C/EBP-*α* reverse, 5′TGTCCAGTTCACGGCTCAGC3′. All reactions involving iTaq SYBR Green Supermix (Bio-Rad Laboratories) were performed using a CFX96 real-time PCR detection system (Bio-Rad Laboratories).

### 2.5. Western Blotting

MSCs treated with hinokitiol were lysed in a lysis buffer (25 mM HEPES (pH 7.4), 100 mM NaCl, 1 mM EDTA, 5 mM MgCl_2_, 0.1 mM dithiothreitol, and protease inhibitor cocktail). Next, cell lysates containing equal amounts of proteins were resolved electrophoretically by performing SDS-polyacrylamide gel electrophoresis on 10%–15% gels, and the resolved proteins were transferred onto nitrocellulose membranes. Immunoreactivity was detected by sequentially incubating the membranes with horseradish peroxidase-conjugated secondary antibodies and by using enhanced chemiluminescence reagents. Images were captured using the Fusion FX7 acquisition system (Vilber Lourmat, Eberhardzell, Germany). Densities of protein bands were analyzed using Bio-1D (Vilber Lourmat, Marne La Vallee, France).

### 2.6. Transmission Electron Microscope (TEM) Analysis

After fixation of samples in 2% glutaraldehyde (EMS, USA) and 2% paraformaldehyde (EMS, USA) in 0.05 sodium carcodylate buffer (pH 7.2) (EMS, USA), specimens were postfixed in 1% osmium tetroxide (EMS, USA) and dehydrated in graded ethanol and propylene oxide (EMS, USA). The cells were embedded in epoxy resin (EMbed-812; NMA, Nadic methyl anhydride; DDSA, dodenyl succinic anhydride; DMP-30) (EMS, USA). Ultrathin sections were cut on an LKB-III ultratome (Leica, Austria) and were stained with 0.5% uranyl acetate (EMS, USA) and lead citrate (EMS, USA). The images were taken on a Hitachi H-7650 electron microscope (Hitachi, Japan) at an accelerating voltage of 100 kV.

### 2.7. Statistical Analysis

All data are expressed as mean ± standard deviation and were compared using Student's *t*-test, analysis of variance, and Duncan's test with SAS statistical software ver. 9.1 (SAS Institute, Cary, NC, USA). Results were considered significant at ^∗^*p* < 0.05, ^∗∗^*p* < 0.001, and ^#^*p* < 0.01.

## 3. Result

### 3.1. Effects of Hinokitiol on the Adipocyte Differentiation of MSCs

**Figure 1:**
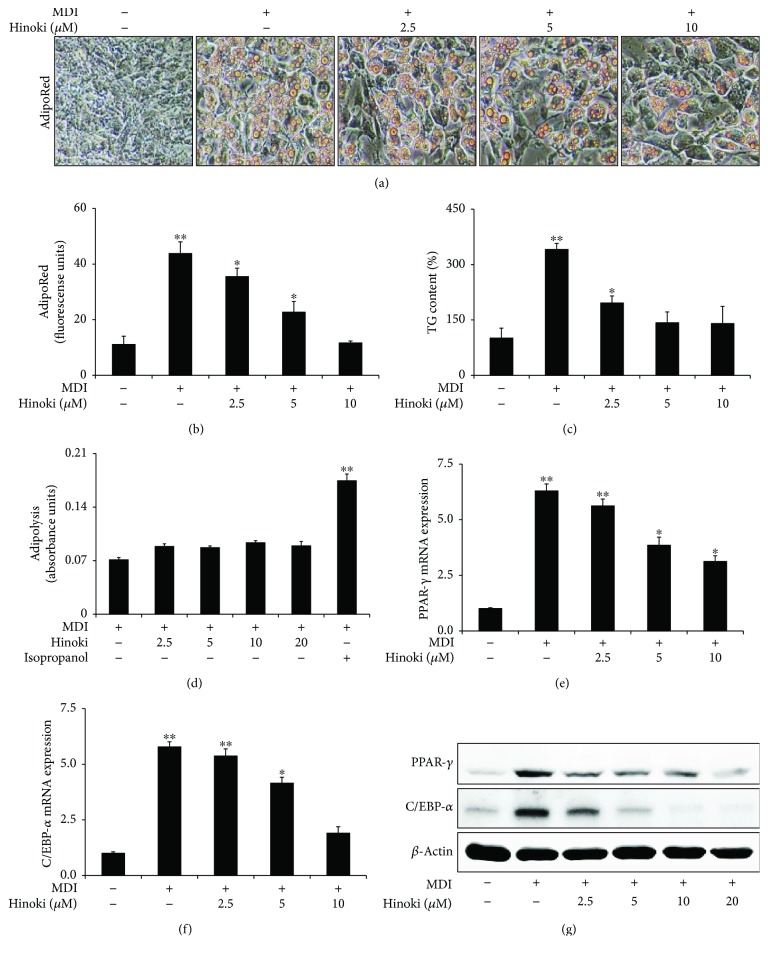
Effect of hinokitiol against adipocyte differentiation in mesenchymal stem cells. MSCs were incubated with hinokitiol at various concentrations (2.5, 5, and 10 *μ*M) following treatment with MDI. The AdipoRed assays were performed on day 6 and were photographed with a light microscope (×200). Fluorescence was measured with an excitation wavelength of 485 nm and an emission wavelength of 572 nm (a and b). Triglyceride (TG) assay (c) and adipolysis (d) were assessed on day 7, and TG contents relative to the control were measured. Total RNA was extracted to quantify the mRNA expression levels of PPAR-*γ* (e) and C/EBP-*α* (f). PPAR-*γ* and C/EBP-*α* proteins were detected by Western blot analysis (g). *β*-Actin was used as loading control. A bar graph was generated using mean ± standard error of the mean (SEM) (*n* = 3). ^∗^*p* < 0.05 and ^∗∗^*p* < 0.01 for significant differences between the control and treatment groups.

First, we confirmed the effects of hinokitiol on adipogenesis in MSCs. We assessed the effects of different concentrations (2.5, 5, and 10 *μ*M) of hinokitiol on the adipocyte differentiation of MSCs by performing the AdipoRed assay (Figures 1(a) and 1(b)). MSCs were preincubated with the MDI medium, followed by incubation with hinokitiol at concentrations of 2.5–10 *μ*M for 7 days. Hinokitiol concentrations above 2.5 *μ*M significantly decreased the adipocyte differentiation of MSCs. Moreover, hinokitiol treatment significantly decreased TG content in MSCs (Figure 1(c)) but did not affect lipolysis (Figure 1(d)). Next, we investigated whether treatment with different hinokitiol concentrations affected the mRNA expression of genes encoding PPAR-*γ* and C/EBP-*α*. PPAR-*γ* and C/EBP-*α* mRNA levels were inhibited by hinokitiol in a dose-dependent manner (Figures 1(e) and 1(f)), consistent with the results of Figures 1(a) and 1(c). Moreover, hinokitiol-inhibited PPAR-*γ* and C/EBP-*α* expression was increased by the MDI medium in a dose-dependent manner (Figure 1(g)).

### 3.2. Hinokitiol Induces Autophagy during the Adipocyte Differentiation of MSCs

**Figure 2:**
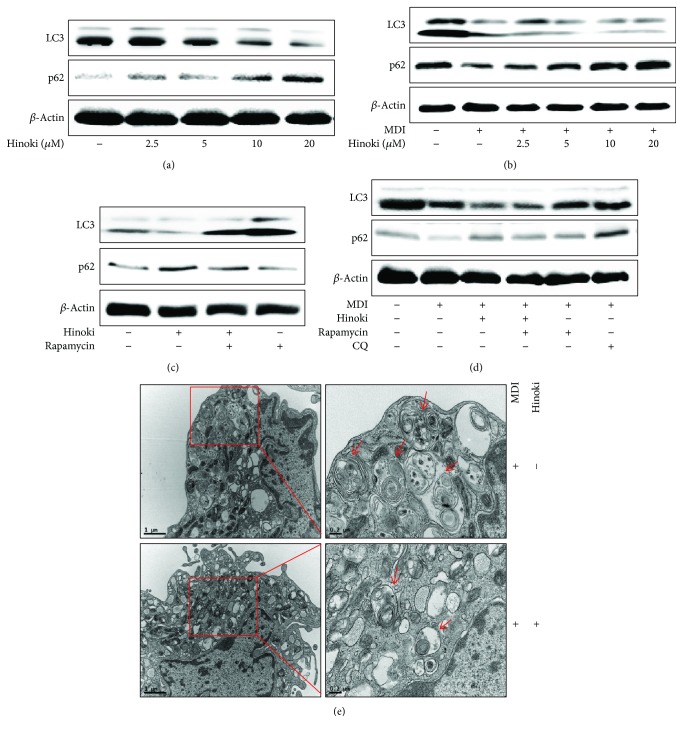
Hinokitiol blocked the autophagy pathway against adipocyte differentiation in mesenchymal stem cells. MSCs in the presence of an autophagy inhibitor (CQ) or autophagy inducer (rapamycin) for 1 h were incubated with hinokitiol at various concentrations (2.5, 5, 10, and 20 *μ*M) following treatment with MDI and harvested at day 2 during the differentiation period. Western blot for LC3 and p62 proteins was analyzed from MSCs. *β*-Actin was used as loading control (a–d). MSCs were incubated with hinokitiol at 10 *μ*M following treatment with MDI for 2 days and analyzed by TEM. Arrowheads indicate autophagosomes (e).

Hinokitiol induces autophagic flux in various cancer cells, including lung adenocarcinoma cells and murine breast and colorectal cancer cells [31, 32]. Therefore, we assessed whether hinokitiol activates autophagy in MSCs and whether hinokitiol-induced autophagy inhibition blocked the adipocyte differentiation of MSCs. First, we measured the levels of LC3-II, a biomarker of autophagy, and p62 in undifferentiated MSCs by performing Western blotting analysis with anti-LC3-II and anti-p62 antibodies (Figure 2(a)). We observed that hinokitiol decreased LC3-II levels but increased p62 levels, which means that autophagy was significantly blocked by hinokitiol. Next, we examined alteration in the levels of various autophagy-regulating proteins in hinokitiol-treated MSCs during adipogenesis (Figure 2(b)). We observed that the p62 level was increased and the LC3-II level was decreased in hinokitiol-treated MSCs compared with that in MDI medium-treated control cells. To determine whether autophagy activation in MSCs is associated with an increase in adipocyte differentiation, we determined the alteration of autophagic flux under enhanced autophagy conditions (Figures 2(c) and 2(d)). For further detection of autophagy induction, we confirmed the presence of autophagosomes using transmission electron microscopy. As shown in Figure 2(e), single-membraned autophagosomes containing organelles were increased by MDI and hinokitiol treatment compared to MDI treatment.

**Figure 3:**
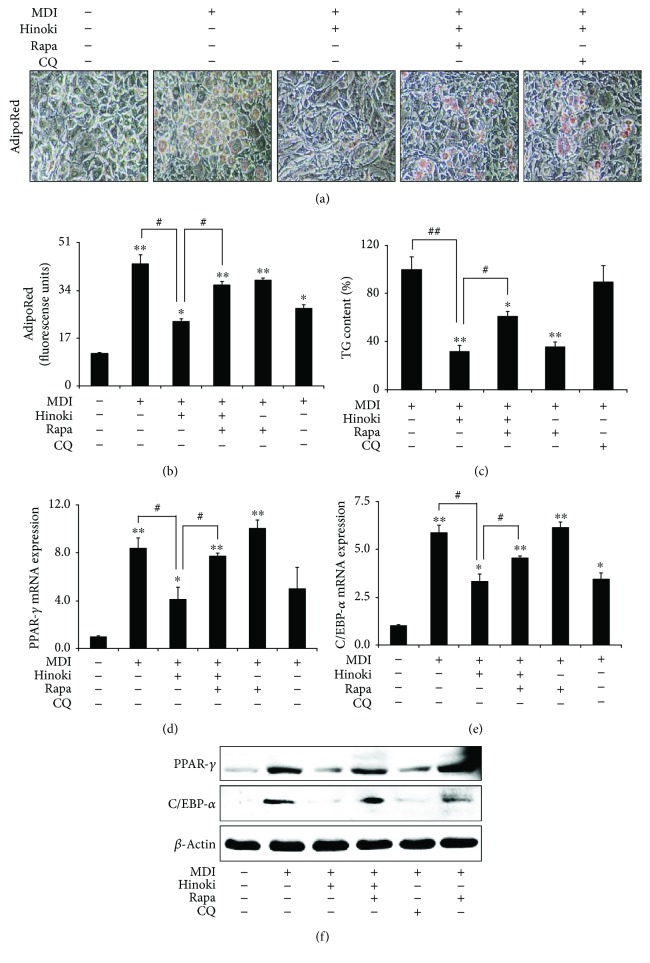
Induction of autophagy restored inhibited adipocyte differentiation by hinokitiol in mesenchymal stem cells. Mesenchymal stem cells (MSCs) in the presence of CQ or rapamycin for 1 h were incubated with 10 *μ*M of hinokitiol, following treatment with MDI. The AdipoRed assays were performed on day 6 and were photographed with a light microscope (×200). Fluorescence was measured with an excitation wavelength of 485 nm and an emission wavelength of 572 nm (a and b). TG assay (c) was assessed on day 7, and TG contents relative to the control were measured. Total RNA was extracted to quantify the mRNA expression levels of PPAR-*γ* (d) and C/EBP-*α* (e). PPAR-*γ* and C/EBP-*α* proteins were detected by Western blot analysis (f). *β*-Actin was used as loading control. Bar graph was generated using mean ± standard error of the mean (SEM) (*n* = 3). ^∗^*p* < 0.05 and ^∗∗^*p* < 0.01 for significant differences between the control and treatment groups and ^#^*p* < 0.05, ^##^*p* < 0.01 for significant differences when compared with the LPS treatment group.

We also examined the effects of hinokitiol on adipogenesis after autophagy induction. For this, MSCs incubated in the MDI medium were pretreated with an autophagy inducer (rapamycin) or inhibitor (CQ) for 1 h, followed by treatment with 10 *μ*M hinokitiol. Hinokitiol treatment prevented MDI medium-induced lipid accumulation. However, these inhibitory effects of hinokitiol on adipogenesis were blocked in cells pretreated with rapamycin (Figures 3(a) and 3(b)). These results were confirmed by measuring TG content with the TG determination kit (Figure 3(c)). Moreover, hinokitiol treatment suppressed MDI medium-induced PPAR-*γ* and C/EBP-*α* mRNA expression (Figures 3(d)–3(e)) and PPAR-*γ* and C/EBP-*α* protein expression (Figure 3(f)). However, pretreatment of MSCs with rapamycin suppressed hinokitiol-induced inhibition of PPAR-*γ* and C/EBP-*α* mRNA and protein expression. These results indicated that hinokitiol suppressed autophagy and differentiation to adipocytes of MSCs.

### 3.3. Hinokitiol Inhibits the Adipocyte Differentiation of MSCs through the AMPK Pathway

**Figure 4:**
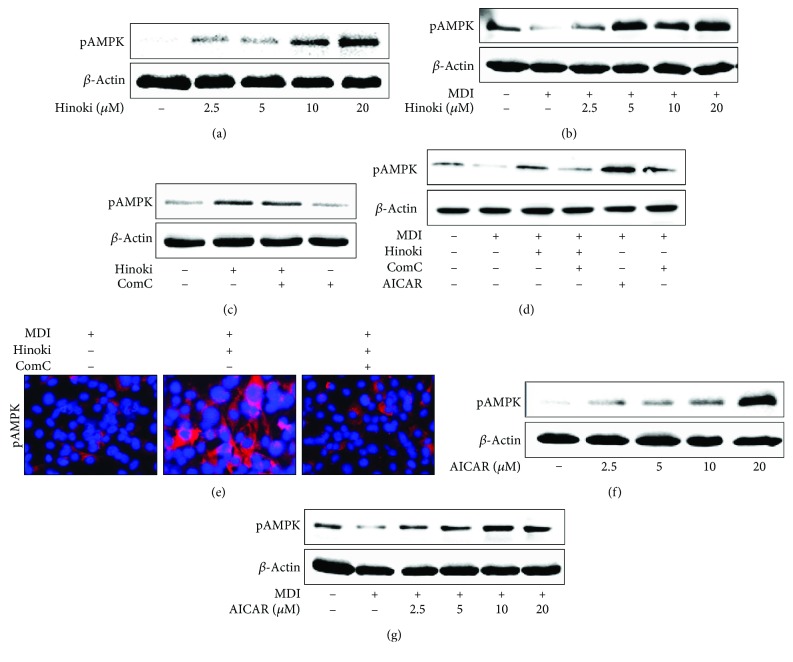
AMPK inactivation restored inhibited adipocyte differentiation by hinokitiol in mesenchymal stem cells. MSCs in the presence of compound C or AICAR for 1 h were incubated with 10 *μ*M of hinokitiol, following treatment with MDI. Western blot for LC3 and p62 proteins was analyzed from MSCs. *β*-Actin was used as loading control (a–d). The cells were immunostained with pAMPK antibody (red) and observed in fluorescent view (e). MSCs were incubated with AICAR at various concentrations (2.5, 5, 10, and 20 *μ*M) following treatment with MDI, and Western blot for LC3 and p62 proteins was analyzed (f, g).

To investigate whether hinokitiol increased AMPK activity in MSCs, we measured phosphorylated AMPK levels in undifferentiated MSCs and MSCs undergoing adipocyte differentiation at concentrations of 2.5, 5, 10, and 20 *μ*M. We observed that hinokitiol increased AMPK phosphorylation in undifferentiated as well as differentiated MSCs in a dose-dependent manner (Figures 4(a) and 4(b)). Next, we treated undifferentiated MSCs with compound C for 1 h, followed by treatment with 10 *μ*M hinokitiol for 24 h. We observed that increased AMPK phosphorylation by hinokitiol was suppressed in MSCs pretreated with the AMPK inhibitor compound C (Figure 4(c)). Similarly, pretreatment of MSCs with compound C suppressed hinokitiol-induced AMPK phosphorylation after incubation with the MDI medium (Figure 4(d)). These data indicated that decreased AMPK phosphorylation by compound C was increased by hinokitiol treatment, suggesting an enhancement effect on the AMPK phosphorylation of hinokitiol. These results indicated that hinokitiol affected AMPK activation, suggesting that AMPK is a possible part of the mechanism of hinokitiol in adipocytes. Next, we investigated whether increased AMPK activation by AICAR upregulated AMPK protein expression, with or without MDI treatment. We observed that AICAR treatment at concentrations of 2.5–40 *μ*M gradually increased phosphorylation of AMPK (Figures 4(f) and 4(g)).

**Figure 5:**
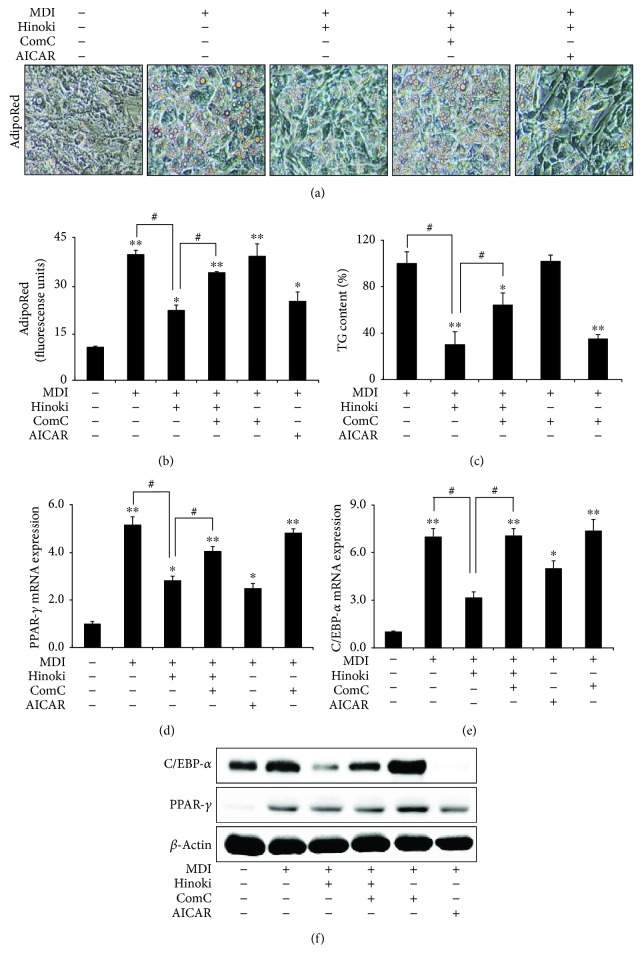
AMPK inactivation restored inhibited adipocyte differentiation by hinokitiol in mesenchymal stem cells. MSCs in the presence of compound C or AICAR for 1 h were incubated with 10 *μ*M of hinokitiol, following treatment with MDI. The AdipoRed assays were performed on day 6 and were photographed with a light microscope (×200). Fluorescence was measured with an excitation wavelength of 485 nm and an emission wavelength of 572 nm (a and b). TG assay (c) was assessed on day 7, and TG contents relative to the control were measured. Total RNA was extracted to quantify the mRNA expression levels of PPAR-*γ* (d) and C/EBP-*α* (e). PPAR-*γ* and C/EBP-*α* proteins were detected by Western blot analysis (f). *β*-Actin was used as loading control. A bar graph was generated using mean ± standard error of the mean (SEM) (*n* = 3). ^∗^*p* < 0.05 and ^∗∗^*p* < 0.01 for significant differences between the control and treatment groups and ^#^*p* < 0.01 for significant differences when compared with the LPS treatment group.

We used AMPK inhibitor compound C to determine whether AMPK activation mediated the effect of hinokitiol on the adipogenesis of MSCs. MSCs incubated in the MDI medium were pretreated with compound C for 1 h, followed by treatment with 10 *μ*M hinokitiol. Pretreatment of MSCs with compound C attenuated the antiadipogenic effects of hinokitiol (Figures 5(a) and 5(b)). Moreover, pretreatment with compound C restored TG levels in matured adipocytes to those in cells incubated with only the MDI medium (Figure 5(c)). We found that inhibition of AMPK phosphorylation blocked the antiadipogenesis effects caused by hinokitiol in the mRNA levels (Figures 5(d) and 5(e)). Moreover, pretreatment of MSCs with compound C suppressed the hinokitiol-induced increase in PPAR-*γ* and C/EBP-*α* protein expression (Figure 5(f)). These results indicated that the hinokitiol-induced antiadipogenesis was restored by AMPK inhibition.

### 3.4. Hinokitiol-Induced Autophagy in Matured Adipocytes Is Mediated by AMPK Activation

**Figure 6:**
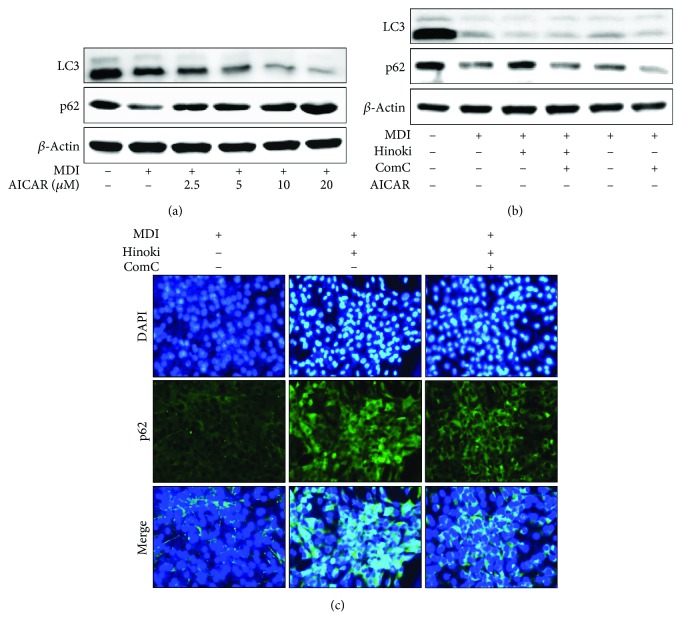
Hinokitiol inhibits adipocyte differentiation through the AMPK pathway in mesenchymal stem cells. MSCs were incubated with hinokitiol at various concentrations (2.5, 5, and 10 *μ*M) following treatment with MDI, and Western blot for LC3 and p62 proteins was analyzed (a). MSCs in the presence of compound C or AICAR for 1 h were incubated with 10 *μ*M of hinokitiol, following treatment with MDI. LC3 and p62 proteins were detected by Western blot analysis (b). *β*-Actin was used as loading control. The cells were immunostained with DAPI (blue) and p62 antibody (green) and observed in fluorescent view (c).

Next, we examined whether hinokitiol-induced autophagy in MSCs was mediated by the activation of the AMPK pathway. MSCs treated with hinokitiol increased phosphorylated AMPK and p62 levels but decreased LC3-II levels. Further, pretreatment of MSCs with the AMPK activator, AICAR, increased AMPK phosphorylation and decreased LC3-II levels in common with that in cells treated with hinokitiol alone (Figure 6(a)). Moreover, we observed that decreased LC3-II levels and increased p62 levels by hinokitiol were reversed by compound C (Figure 6(c)). These results indicated that incubation of MSCs with the MDI medium, followed by hinokitiol treatment, significantly decreased autophagy activation through the AMPK pathway. Therefore, we concluded that the activation of the AMPK pathway decreased autophagy in MSCs and that hinokitiol prevented the adipocyte differentiation of MSCs by activating the AMPK pathway and by inhibiting autophagy.

## 4. Discussion

Hinokitiol is a natural tropolone-related compound, isolated from the wood of *Chamaecyparis taiwanensis* [33]. Hinokitiol exerts various biological and pharmacological effects [34], including antimicrobial [35], antibacterial [36], neuroprotective [37], anticancer, and anti-inflammatory effects [32, 38]. However, the effect of hinokitiol on adipocyte differentiation is unknown.

MSCs are multipotent progenitor cells that can differentiate into different cells such as adipocytes, chondrocytes, osteoblasts, and myocytes [39–42]. Under conventional culture conditions [43], MSCs differentiate into adipocytes [44] through a process that can be divided into commitment stage and terminal differentiation stage [45, 46], and they are appropriate for performing studies on human adipogenesis [47]. In the present study, we induced the differentiation of MSCs to adipocytes and examined adipogenesis-related factors.

Adipogenesis involves multiple steps and is regulated by several transcription factors. PPAR-*γ* and C/EBP-*α* play crucial roles as major transcription factors in adipogenesis [9] and induce the expression of several genes involved in the differentiation and maturation of MSCs into adipocytes.

When AMPK activation occurs, AMPK plays a role as a cellular energy sensor and modulates cellular metabolism [48]. In addition, AMPK functions as a metabolic regulator of energy homeostasis with a role in metabolism, including glucose uptake, fatty acid *β*-oxidation, lipolysis, and adipokine secretion [49], and a role in metabolic disorders such as obesity [50] and type 2 diabetes [51]. Moreover, previous studies have reported that AMPK activation blocks adipogenesis [52, 53] by downregulating the expression of several adipocyte-specific transcription factors [54]. Therefore, in the present study, we examined whether hinokitiol regulates adipogenesis leading to inhibit adipogenesis by AMPK activation.

Autophagy is a unique protein degradation pathway through which cytoplasmic constituents are delivered to the lysosome for digestion. Autophagy plays a beneficial role in providing energy by recycling macromolecules in response to nutrient and environmental factors [55–57]. Moreover, autophagy may be critical for normal adipogenesis and maintenance of adipose tissue homeostasis [15, 17, 58]. However, mechanisms underlying the regulation of autophagy in adipocytes by AMPK signaling are unclear. Studies have reported that hinokitiol induces autophagy by inhibiting the proliferation in various cancer cell lines [31, 59, 60]. In the present study, we confirmed that hinokitiol inhibits autophagy in adipocytes.

The results of the present study indicated that hinokitiol significantly increased AMPK phosphorylation to produce active AMPK and suggested that hinokitiol inhibited the adipocyte differentiation of MSCs.

## Data Availability

The data used to support the findings of this study are available from the corresponding author upon request.

## Abbreviations

AMPK: AMP-activated protein kinase
PPAR-*γ*: Peroxisome proliferator-activated receptor-*γ*
MSCs: Mesenchymal stem cells
C/EBP: CCAAT enhancer-binding proteins
TG: Triglyceride.
